# Supplementary material for: The interactome of intact mitochondria by cross-linking mass spectrometry provides evidence for coexisting respiratory supercomplexes
Source: Mol Cell Proteomics. 2017 Dec 8;17(2):216–32. doi: 10.1074/mcp.RA117.000470 (PMC5795388; doi:10.1074/mcp.RA117.000470)
Supplement: Supplemental Data [file supp_RA117.000470_133922_0_supp_23978_fzffwf.zip › spectra_annotation/mito_spectra_annotation/32-1-2-1-2-1.pdf]

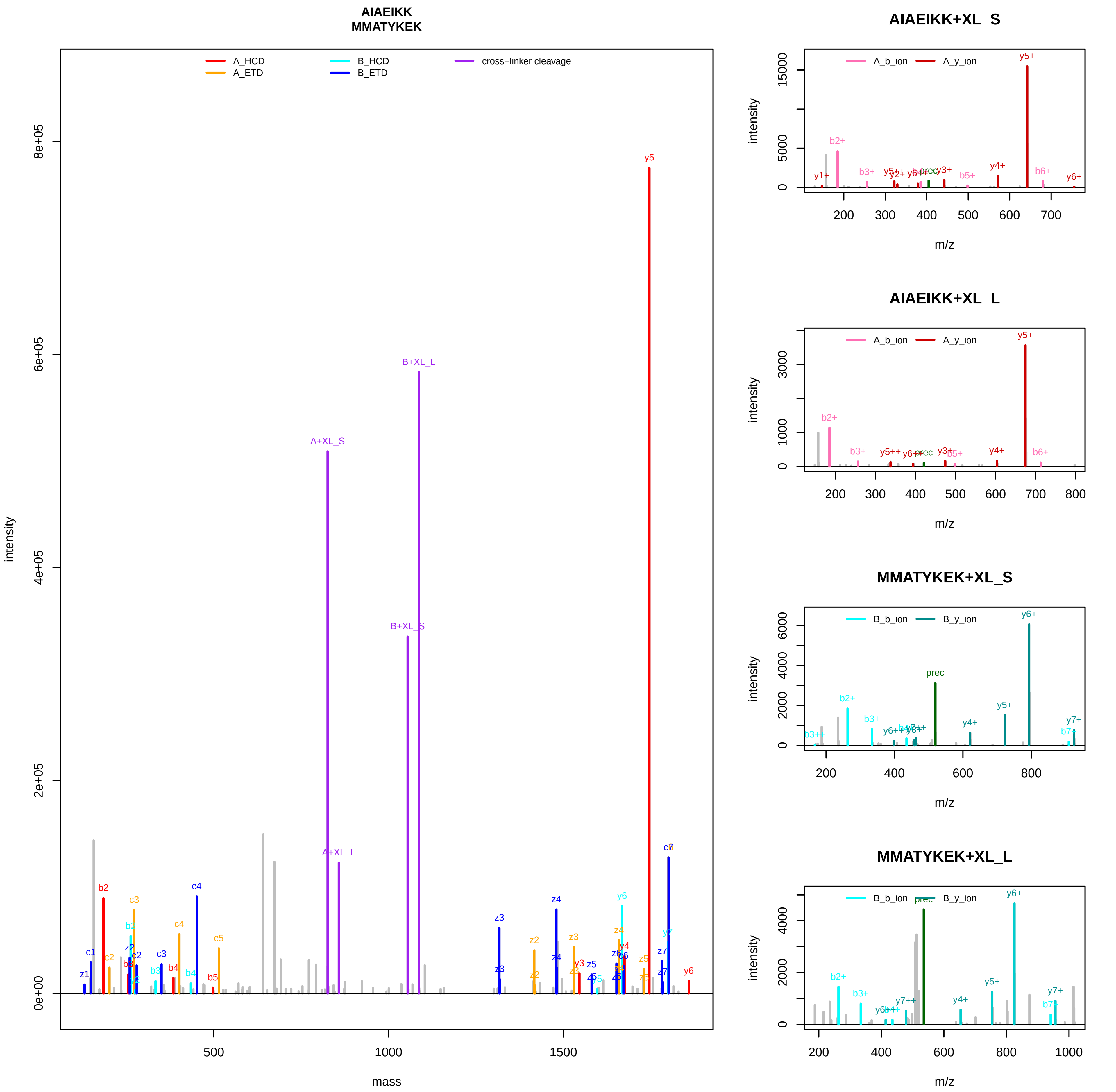
AIAEIKK
MMATYKEK
A_HCD
A_ETD
B_HCD
B_ETD
cross−linker cleavage
intensity
mass
0e+00
2e+05
4e+05
6e+05
8e+05
500
1000
1500
y5
B+XL_L
A+XL_S
B+XL_S
A+XL_L
b2
c3
c4
c5
b4
b5
z3
z4
z2
y3
y4
y6
c7
z7
z5
y7
AIAEIKK+XL_S
A_b_ion
A_y_ion
y5+
b2+
b3+
b5+
b6+
y1+
y3+
y4+
y6+
prec
m/z
AIAEIKK+XL_L
MMATYKEK+XL_S
B_b_ion
B_y_ion
y6+
y5+
y4+
y7+
b2+
b3+
MMATYKEK+XL_L
